# Long-acting paliperidone in Ekbom’s syndrome in Lewy body dementia: A case report

**DOI:** 10.1515/tnsci-2022-0230

**Published:** 2022-07-28

**Authors:** Laura Orsolini, Diana Corona, Virginio Salvi, Umberto Volpe

**Affiliations:** Department of Clinical Neurosciences/DIMSC, Unit of Clinical Psychiatry, Polytechnic University of Marche, 60126, Ancona, Italy

**Keywords:** delusional parasitosis, Ekbom syndrome, long-acting injectable antipsychotic, LAI, paliperidone, paliperidone palmitate

## Abstract

**Introduction:**

Ekbom Syndrome (ES) is characterised by fixed, delusional beliefs that one’s body is infested by parasites or other vermin in absence of supporting clinical evidence. Antipsychotic (AP) treatment, including long-acting injectable (LAI) AP in subjects with poor compliance, is prescribed to manage behavioural and psychotic symptomatology.

**Objectives:**

We describe a 70-year-old woman who was hospitalised after experiencing new-onset delusions of infestation with visual and tactile hallucinations that led to bizarre behaviours and progressive social withdrawal.

**Methods:**

She was diagnosed with ES and was initially treated with risperidone 3 mg; however, due to poor compliance and a lack of insight, she was switched to LAI palmitate paliperidone (LAI-PP). She was followed up for 8 months, administering Positive and Negative Syndrome Scale, Montreal Cognitive Assessment, Global Assessment of Functioning, Brief Psychiatric Rating Scale, neurocognitive assessment, and neuroimaging.

**Results:**

After a progressive cognitive deterioration, she was diagnosed with an ES secondary to Lewy body dementia (DLB).

**Conclusion:**

The LAI-PP treatment determined a complete clinical remission of psychotic symptoms despite the emergence of an iatrogenic akinetic-rigid syndrome. The delay of confirmatory neurological diagnosis, the associated risky behaviours of the patient, and poor treatment adherence led clinicians to prescribe LAI-PP following a good clinical response to oral paliperidone. However, in the case of a suspected DLB diagnosis, the prescription of an LAI-PP as a first-line strategy should be carefully evaluated.

## 1 Introduction

Ekbom syndrome (ES), also known as delusional parasitosis, is a term that defines a group of neuropsychiatric disorders characterised by the unwavering, fixed, delusional belief that one’s body or immediate environment is infested by parasites, insects, other vermin, or even unanimated materials [1], which is usually accompanied by abnormal cutaneous sensations such as itching, tingling, formication, or pain [2]. These tactile hallucinations are explained with subjective certainty through the aforementioned infestations and often lead to self-mutilation through erosions, excoriations, obsessive cleaning, or even burns and cuts, which represent the subject’s attempt to remove the fictitious parasites [3]. Patients dedicate a lot of time and effort attempting to demonstrate the existence of the pathogens, which drives them to seek help from many physicians, particularly dermatologists and infectivologists, as well as clinical laboratories [3]. Because they are unable to criticise the delusion despite the lack of clinical evidence to support their claims, there is a significant delay in first contact with a psychiatrist and initiating psychopharmacological therapy [1].

ES is a relatively rare condition, with an estimated incidence of 1.9 in 100,000 person-years [4], though more recent studies suggest a prevalence of 27.3 per 100,000 person-years. It seems to occur more frequently in middle-aged and older women [5]. In 14.4% of cases, it might present as a *Folie a deux*, a condition in which the delusion of one individual affects another close to them, usually a family member, to the point where both share the same delusional ideation [6]. ES might occur as a primary delusional disorder, but it may as well be related to substance use or present as a secondary condition to other disorders, namely psychiatric, somatic, and iatrogenic [7]. It can most often result from schizophrenia, dementia, depression, diabetes, neuropathies, and cardiovascular disorders [7].

Overall, the literature suggests that ES should be managed by treating the core symptomatology with antipsychotics (APs) and, when applicable, addressing the underlying illness with adequate therapy in the case of secondary ES [1]. Due to the aforementioned features of rarity and elusiveness of the condition, the literature lacks controlled trials, and thus response rates for AP treatment are yet to be established, as evidence is currently restricted mostly to systematic reviews and heterogeneous case reports [8]. Even though studies have shown no clinically relevant differences in the effectiveness between treatment with first-generation APs and second-generation APs (SGA), current medical practice and the most recent studies suggest using SGA due to their safety and tolerability profile [9]. Despite the intrinsic lack of illness awareness and subsequent poor compliance to therapy, patients with ES have a good prognosis and remission of all florid psychotic symptoms when treated with continuity, with success rates ranging from 70 to 75% [9,10]. Therefore, APs in long-acting injectable (LAI) formulations could represent a preferred treatment option in ES due to their lower occurrence of adverse effects compared to oral counterparts as well as the guarantee of better treatment adherence [1].

In this case, we describe a patient who presented with ES, lately diagnosed with dementia with Lewy bodies (DLB). DLB is the second most common cause of degenerative dementia, but it is still underdiagnosed [11]. It may present itself as a variety of clinical pictures with symptoms involving sleep pattern, motor functions, cognitive impairment, cholinergic deficiency, and neuropsychiatric symptoms [11,12] – the latter typically consist of visual hallucinations (identified as one of the core symptoms for DLB diagnosis [13]) and delusions, as well as changes in mood and affect, more frequently with depressed mood, anxiety, and apathy. Hallucinations are reported by 50% of the patients suffering from LBD. They usually appear in the early stages as visual hallucinations that manifest as recurring, well-formed complex images, in contrast to schizophrenia spectrum disorders, where early-stage hallucinations are predominantly non-visual [14]. It is not uncommon that patients with undiagnosed DLB may present with late-onset psychiatric symptoms, with these symptoms potentially preceding the DLB diagnosis by many years, as described in prodromal DLB, posing a challenging differential diagnosis for clinicians [15]. Identification of the underlying cause is especially crucial in these presentations, as DLB – and, in general, dementia patients – typically shows neuroleptic sensitivity, which poses a high risk of developing important adverse reactions if psychiatric symptoms are treated as usual [16]. In these cases, the presence of other core symptoms, such as rapid eye movement (REM) sleep disorder or parkinsonism, may offer a useful hint, whereas cognitive impairment may prove difficult to evaluate as acute psychotic symptoms could act as a confounding factor [15].

ES is a rare but possible presentation of DLB [17], yet very few cases have been reported [18,19] and different therapeutic approaches have been further investigated. In particular, in one case [18], a patient was initially treated for depression, and then developed an olothymic ES. This event, associated with parkinsonism and cognitive deterioration, led to a DLB diagnosis. She was treated with an association of mirtazapine and low-dose aripiprazole, and when the psychotic symptoms relapsed with therapy suspension, she was treated with a synergic association of donepezil and low-dose aripiprazole, which resulted in ES remission. Aripiprazole was chosen in light of its D_2_ partial agonist action, which is connected to a reduced risk of extrapyramidal symptoms, despite the literature showing no specific benefit. In another case [19], a patient with depression and ES was treated with citalopram and rivastigmine, with the improvement of positive symptoms. In both these cases, DLB was diagnosed before treatment choice, allowing them to avoid treatment that could potentially heighten the risk of neuroleptic sensitivity.

## 2 Patient information

We describe the case of a 70-year-old Caucasian woman who presented to the Emergency Department (ED) with new-onset delusions of infestation associated with pervasive visual and tactile hallucinations that significantly impaired her behaviour. This symptomatology had emerged about 9 months prior to the hospitalisation with an exaggerated preoccupation regarding the possibility that her home could be infested by insects. Therefore, she promptly called several disinfestation services to solve the problem. These behaviours were initially not so evident to her friends as she progressively withdrew socially, also due to the COVID-19 lockdown. Progressively, she started to experience a plethora of symptoms that became extremely pervasive, particularly during the month before her hospitalisation, with the emergence of delusions and hallucinations associated with progressive social withdrawal and the emergence of potentially risky behaviours for her health due to the various disinfestations. In fact, she was so absorbed by overcleaning, disinfecting, and numerous attempts of disinfestation of her home, clothes, and vehicle that she had a car accident – albeit with no repercussions on her or anyone else’s health – as she was trying to clear the windscreen from these hallucinatory insects whilst driving. The patient had no previous history of psychiatric or substance use disorder, but she had been experiencing recently (less than 1 month) occurring sporadic episodes of amnesic aphasia. At the time of her hospitalisation, she had only a previous medical history of systemic hypertension that had been adequately treated with atenolol. Prior to the previous 9 months, she had reportedly shown no sign of social or psychological impairment. She had been a teacher for 35 years and married for 17 years, until her husband died 9 years before.

A full description of a clinical case of ES has been provided here, in accordance with the CARE (CAse REport) Statement, Checklist and Guidelines [20]. The patient described herein gave written consent for the publication of the presented findings. She was monitored through consecutive clinical examinations, clinical and neurocognitive assessment tests, and neuroimaging examinations, as well as routine blood tests, in accordance with a precise diagnostic flowchart previously proposed by our research team [1] (Table 1). The patient was observed for an overall period of 8 months. She was first evaluated at the moment of admission (*T*_0_), then again after 2 weeks at the time of discharge from the hospital (*T*_1_); furthermore, she was monitored through a follow-up starting 1 month after discharge (*T*_2_), consisting of monthly appointments (*T*_2_–*T*_8_, with an exception where *T*_**7**_ occurred 2 months after *T*_6_).

**Table 1:** Timeline of clinical assessment

	Rating scales	Type of assessment	*T*_0_	*T*_1_	*T*_2_	*T*_3_	*T*_4_	*T*_5_	*T*_6_	*T*_7_	*T*_8_
Clinical assessment	BPRS	General psychiatric illness	X	X	X	X	X	X	X	X	X
	GAF	General functioning	X	X	X	X	X	X	X	X	X
	CGI	Global Progress	X	X	X	X	X	X	X	X	X
	PANSS	Psychotic symptoms	X	X	X	X	X	X	X	X	X
Neurocognitive assessment	MoCA	Cognitive functions	X	X	X	X	X	X	X	X	X
	MMSE	Cognitive functions in the elderly		X		X			X	X	
	RPM	Fluid intelligence – logic and deduction		X					n/a		
	SCWT	Selective attention		X					n/a		
	LURIA	Executive functions		X					X		
	DGS	Verbal and working memory		X					n/a		
	RAVLT	Short- and long-term memory		X					X		
	CORSI	Memory span		X					n/a		
	ROCF	Visuospatial abilities		X					X		
	APRAXIA	Ideomotor apraxia		X					X		
	VFT	Lexical competence		X					X		
	DENOMINATION	Lexical competence		X					X		
	PGT	Agnosia differential diagnosis		X					X		
	FAMOUS	Aphasia		X					n/a		

Abbreviations: BPRS = Brief psychiatric rating scale; GAF = Global assessment of functioning; CGI = Clinical global impression; PANSS = Positive and negative syndrome scale; MoCA = Montreal cognitive assessment; MMSE = Mini mental state examination; RPM = Raven’s progressive matrices; SCWT = Stroop colour and word test; LURIA = Luria’s sequence; DGS: Digit span; RAVLT = Rey-auditory verbal learning test; CORSI = Corsi test; ROCF = Rey–Osterrieth complex figure; APRAXIA = Apraxia test; VFT = Verbal fluency test; Denomination = Denomination upon description; PGT = Poppelreuter–Ghent’s overlapping figures test; FAMOUS = Famous face recognition and naming test.

**Figure 1:**
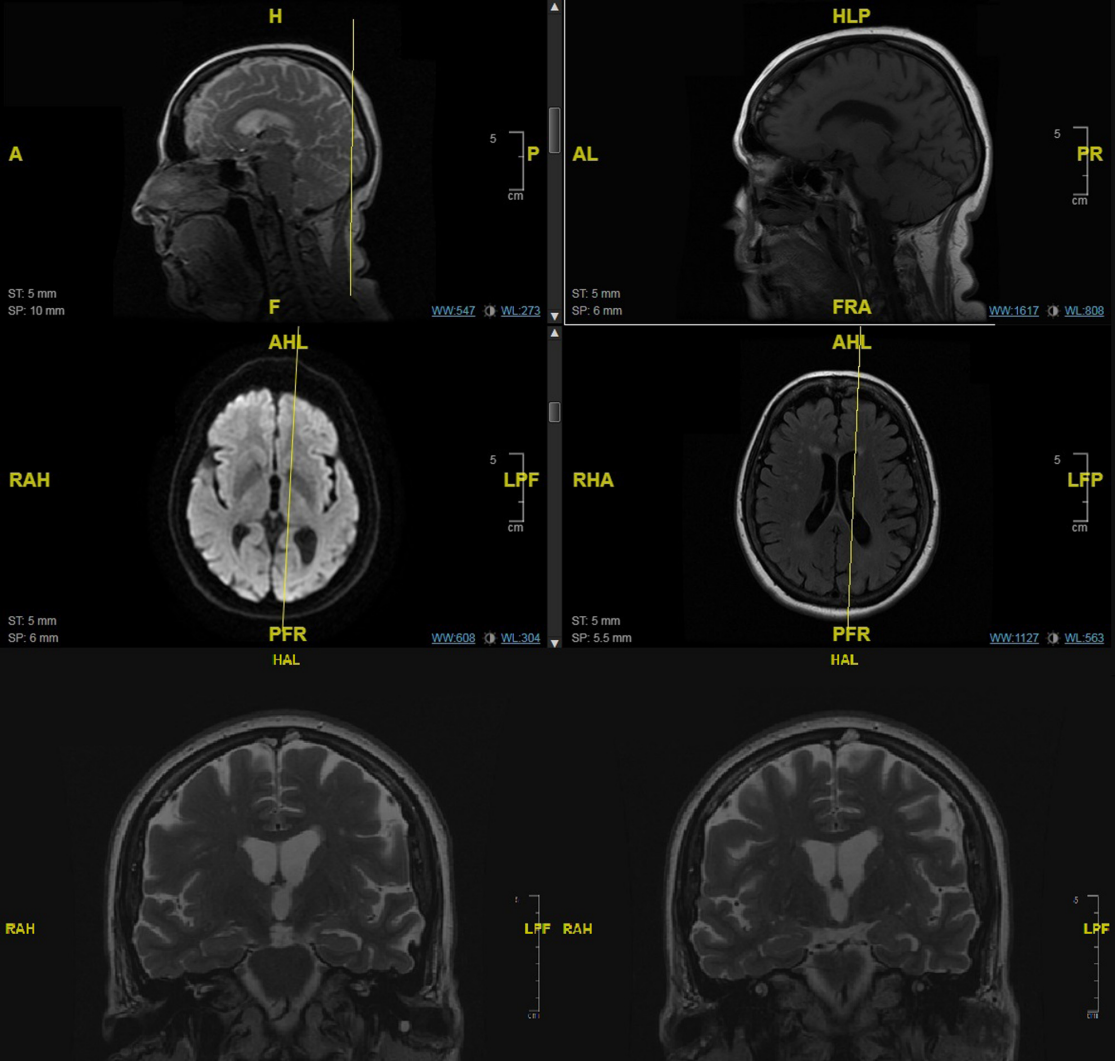
Brain MRI of the patient. The imaging was obtained at *T*_6_ and shows global alterations suggesting a primary neurodegenerative condition. Relative preservation of medial temporal lobe volume can be observed.

**Figure 2:**
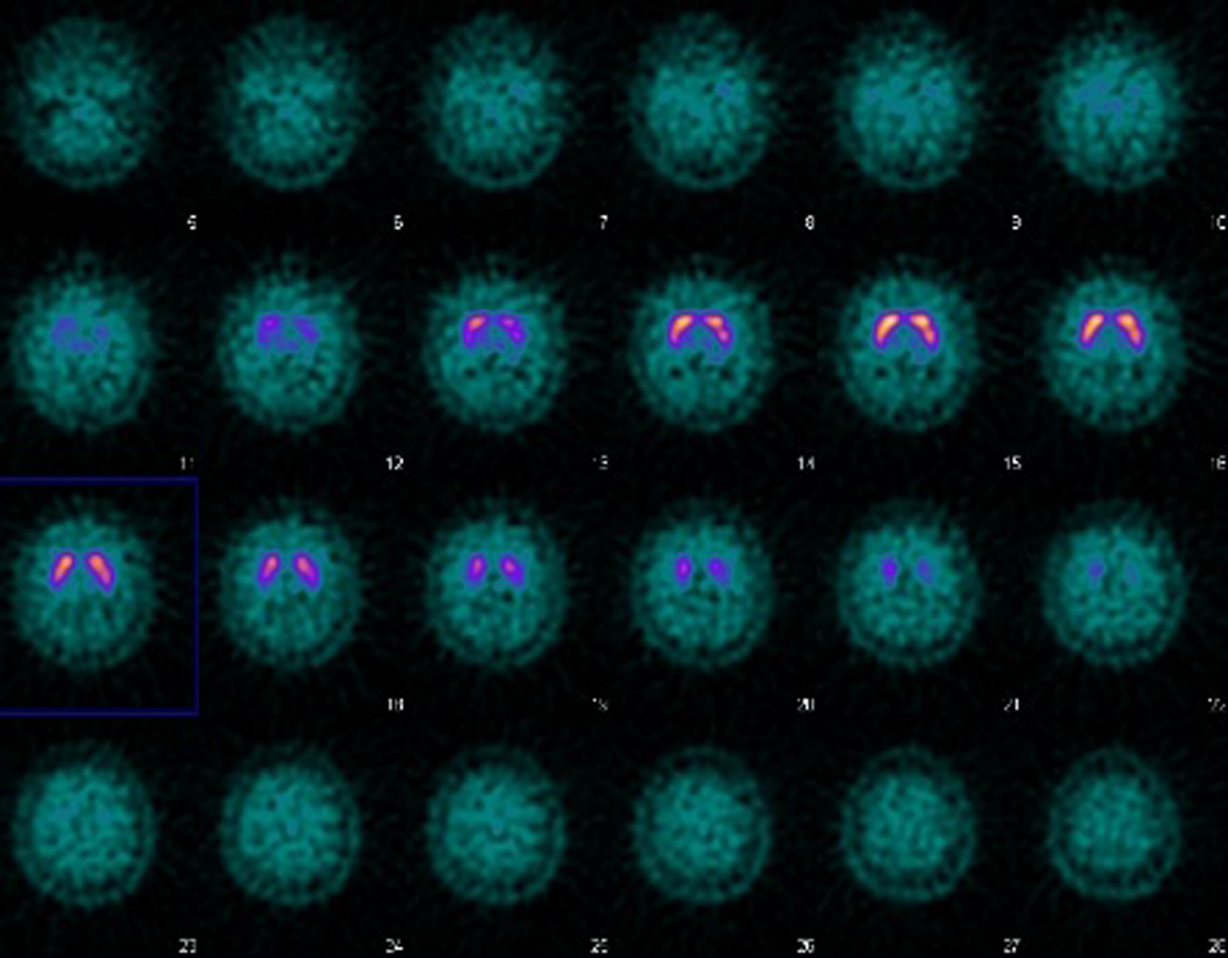
DaT Scan of the patient. The imaging was obtained between *T*_7_ and *T*_8_.


Moreover, following the neurologist’s consultation, further neuroimaging studies were performed during the 6-month follow-up period, including a brain magnetic resonance imaging (MRI) (Figure 1) and a dopamine transporter scan (DaT Scan) (Figure 2).

**Ethical approval:** The research related to human use has complied with all relevant national regulations, institutional policies, and the tenets of the Helsinki Declaration and has been approved by the authors’ institutional review board or equivalent committee.
**Informed consent:** Informed consent has been obtained from all individuals included in this study.

## 3 Timeline

### 3.1 Psychiatric Hospitalisation (*T*_0_–*T*_1_)

The patient was admitted (*T*_0_) to the psychiatric ward following a psychiatric consultation taken during her admission to the ED, where she was accompanied by a friend who is also a physician. At the time of her admission, she manifested delusional ideation of infestation and somatic delusions, along with microzoopsies and tactile hallucinations. She experienced high levels of distress and a depressed mood with labile affect, sometimes with inappropriate affectivity. Moreover, a clinical examination revealed signs of initial cognitive impairment. The clinical picture led to the suspicion of an ES, as the possibility of actual skin infestation had already been excluded in the ED setting, and during the physical exam, the patient showed no signs of parasitosis or blood alterations that might have suggested such conditions. Full laboratory blood tests were also performed to exclude other common causes of secondary ES, such as endocrinological conditions and vitamin deficiencies, but evidenced the presence of a monoclonal gammopathy, which was then investigated further to identify a possible malignant cause of the psychiatric symptoms. Certain forms of cancer, in fact, may cause chronic pruritus, which is suspected to act as a precipitating factor in ES [21]. A haematology consult excluded the presence of malignant blood neoplasia. A brain computerised tomography (CT) was also performed to exclude the signs of acute cerebral diseases. At the time of admission (*T*_0_), she was administered Positive and Negative Syndrome Scale (PANSS) [22,23], Montreal Cognitive Assessment (MoCA) [24,25], Clinical Global Impression [26], Global Assessment of Functioning (GAF) [27], and Brief Psychiatric Rating Scale (BPRS) [28,29,30] (Table 2).

**Table 2:** Scores of clinical assessment

	*T*_0_	*T*_1_	*T*_2_	*T*_3_	*T*_4_	*T*_5_	*T*_6_	*T*_7_	*T*_8_
PANSS+	26	15	23	12	9	8	10	7	9
PANSS−	9	9	19	24	28	31	29	28	21
PANSS	94	62	86	78	71	80	67	71	58
MoCA	14	14	14	13	8	8	6	6	3
CGI	7	6	4	4	4	3	3	3	4
GAF	30	55	45	50	35	40	45	45	60
BPRS	66	40	57	52	44	54	46	36	29

Abbreviations: PANSS+ = Positive scale; PANSS− = Negative scale.

As the diagnosis of ES was confirmed, the patient was initially treated with AP therapy consisting of oral risperidone up to 3 mg per day – the usual first-line recommendation for these patients [31]. This treatment led to an improvement of mood and affect, accompanied by a partial remission of positive symptomatology at *T*_1_ (Table 2). Contextually, following subsequent interviews with her caregivers and friends, a series of further investigations were planned to pose a differential diagnosis and to exclude cognitive deterioration or an organic condition causing the ES. This included a first neurologist examination that suggested a neurocognitive assessment, aiming to assess deficits in different cognitive functions through a battery of tests (mini mental state examination [MMSE] [32,33], Raven’s progressive matrices [34,35], Stroop colour and word test [36], Luria’s sequence [37], digit span [38,39], Rey-auditory verbal learning test [40,41], Corsi test [42,43,44], Rey–Osterrieth complex figure [45,46], apraxia test [44], verbal fluency test [38], DENOMINATION [47], Poppelreuter–Ghent’s overlapping figures test [48,49], famous face recognition and naming test [50]). These preliminary evaluations were first performed during the psychiatric hospitalisation (Table 1) in a clinical picture characterised by a florid and severe psychotic symptomatology, which may have masked the effective neurocognitive impairment at the time. At the first assessment, CT was able to rule out masses or major events that could be suspected in such a late-onset presentation, but both neurocognitive assessment and brain CT showed only unspecific evidence, not necessarily compatible with early signs of dementia, so the neurology consult suggested to proceed with AP therapy and longitudinal monitoring to allow for better evaluation once positive symptoms would not affect the neurologic examination. For this reason, a brain MRI and a second neurological evaluation were planned after discharge from hospital.

The patient was then referred to the Regional Department of Mental Health for standard patient care at the local public clinic. However, due to the complexity of the clinical case and the need to follow up the patient for a longitudinal differential diagnosis for excluding/confirming a neurological condition, the patient and her caregiver agreed to perform a monthly follow-up at our hospital outpatient clinic, consisting of a multidisciplinary approach with an evaluation of both psychiatric and neurological symptoms. Moreover, due to poor treatment compliance, poor illness insight, and awareness, it was established in agreement with the referring psychiatrist to switch from oral treatment to paliperidone palmitate in LAI formulation (PP-LAI). Therefore, she began the LAI treatment with the induction phase, consisting of a first intramuscular injection of 150 mg of PP-LAI, followed by a second intramuscular injection of 100 mg 7 days later. The treatment led to a substantial symptomatology remission, as reported by the improvement on the positive scale at PANSS starting as early as *T*_1_. At this phase, she was discharged with a diagnosis of Acute polymorphic psychotic disorder with symptoms of schizophrenia [F 23.1], as defined by the International statistical classification of diseases and related health problems (10th ed., ICD-10, World Health Organization, 2016), and a suggested therapy consisting of a monthly injection of PP-LAI at a dosage of 75 mg.

### 3.2 First follow-up period (*T*_2_–*T*_4_)

At the first follow-up appointment (*T*_2_), a general psychopathological relapse was documented (Table 2), along with a global functioning impairment; moreover, the patient had refused to take the third injection of PP-LAI the day before the follow-up. Due to the lack of insight and recurrent positive and negative symptomatology, she was administered PP-LAI through an involuntary treatment a week later. Subsequent follow-up visits evidenced a constant improvement of positive symptoms with good compliance and satisfactory response to PP-LAI, leading to a complete remission of the florid psychotic disorder (*T*_3_–*T*_4_). Concurrently, the patient showed a worsening motor impairment and thought retardation, hypomimia, blunted affect, and emotional withdrawal. Moreover, after 3 months of PP-LAI treatment (*T*_4_), she experienced an extrapyramidal akinetic-rigid syndrome [51,52] characterised by bradykinesia, postural tremor, and Pisa syndrome [53]. These symptoms determined a significant negative impact on her general physical and mental status, as well as impaired socio-personal functioning, as observed at the BPRS total scores, PANSS Negative subscale, and progressively lower GAF scores from *T*_2_ to *T*_5_. The quick onset of the abovementioned adverse effects, together with a concomitant, rapid, and progressive cognitive decline, posed the strong suspicion of a neuroleptic sensitivity that is typically associated with an underlying neurodegenerative illness [54,16,18]. This suspicion was managed through specialistic neurological consultations, which initially prescribed folic acid supplements and statins, and then suggested the administration of memantine (*T*_3_–*T*_4_), which was immediately discontinued due to the development of side effects (e.g. increased agitation, irritability, and restlessness). Simultaneously, the brain MRI (Figure 1) documented frontal atrophy and an altered signal in occipital areas, thus supporting the initial hypothesis of a neuro-organic disease underpinning the ES.

### 3.3 Second follow-up period (*T*_5_–*T*_8_)

At *T*_5_ and *T*_6_, the patient was recommended to gradually discontinue PP-LAI treatment due to the occurrence of the abovementioned adverse effects; the last injection of PP-LAI was administered 2 weeks after *T*_5_. However, despite PP-LAI discontinuation, the improvement of psychotic symptoms was maintained over time, as shown by the Positive subscale of the PANSS from *T*_6_ to *T*_8_, and an overall improvement in general functioning was obtained, as shown from the scoring at GAF. The Negative subscale of PANSS over the same period showed only little symptomatology remission; the improvement was less substantial than expected; in fact, patient improved the secondary negative symptomatology induced by the PP-LAI following its discontinuation, but at the same time the scores were significantly raised by the occurrence of central neurocognitive degeneration signs. This decline was shown through a self-reported dramatic worsening of functional independence in everyday tasks, coherently with monthly MoCA and MMSE total scores. This evidence posed the necessity of a differential diagnosis, so a neurocognitive battery was requested. This second complete neurocognitive assessment was repeated at *T*_6_, after 6 months from *T*_0_ (Table 1), with the aim to longitudinally monitor cognitive deterioration status. The evaluation confirmed the suspicion of a rapidly progressive cognitive deterioration with impairment in executive functions and visuospatial abilities and compromised autonomy.

Furthermore, following the neurologist’s consultation, a DaT Scan was requested in order to exclude Parkinson’s disease, which found no substantial alteration of the nigrostriatal dopaminergic system, confirming that the extrapyramidal symptoms was exclusively due to an iatrogenic parkinsonism from the administration of PP-LAI injections (Figure 2). This result was obtained at *T*_8_ and was subsequently followed by a further neurology consultation that, in consideration of the presence of cognitive deterioration with impairment in executive functions and visuospatial abilities, a core symptom (visual hallucinations) and late-onset supporting clinical features (delusions, depressed mood, and hallucinations in other modalities) posed the diagnosis of possible DLB (as of the 2017 revised diagnostic criteria [13]). Given the negative DaT scan, the criteria for a plausible DLB were not met, so at this stage, it could have been appropriate to investigate further the presence of the other two indicative biomarkers that could have met the criteria: the patient could have been assessed through an Iodine-123 metaiodobenzylguanidin myocardial scintigraphy, which typically shows reduced uptake in DLB subjects, or through a polysomnography, which could have evidenced REM sleep without atonia. Given the complete remission of psychotic symptomatology and the neurologic diagnosis, follow-up with the psychiatric clinic was suspended and the continuity of care for the case was handed over to the local dementia outpatient service.

## 4 Discussion

The present case report described a new-onset ES in a woman without a previous psychiatric history, which was effectively and rapidly managed with risperidone oral treatment. Given the poor treatment adherence and the high chance of full remission if adequately treated with APs, it was chosen to switch to an LAI formulation, as recommended by the literature [1]. PP-LAI was preferred, due to the positive clinical response achieved with oral risperidone and its good tolerability profile. In this case, PP-LAI assured continuity in pharmacotherapy, which led to a stable remission of delusions and hallucinations, as confirmed by all clinical assessments and examinations over the follow-up observation. Regardless of PP-LAI discontinuation due to the onset of adverse effects, the patient did not manifest a relapse of positive symptoms over the entire 8-months of clinical observation.

There are few studies investigating the pharmacologic approach to ES [6,7,9,55,56,57,58], with most studies suggesting treatment with SGA, mainly due to their acceptable safety and tolerability profile [1,59]. In general, the choice of the type of SGA should mainly be derived from their tolerability profile and the risk of concomitant pharmacological interactions. The choice to prescribe an LAI AP should yet be fully confirmed in ES. In fact, only a few case reports have been published so far [60,61]. Therefore, the use of LAI AP therapy in patients with ES is still under investigation, although it may represent a promising therapeutic approach, particularly in those subjects with poor treatment adherence and compliance. That is specifically valuable for ES patients who often tend to avoid contact with psychiatric services, despite the fact that AP may often be successful and able to provide the patient with a good prognosis with a full clinical remission [1]. The psychiatric symptomatology remission and persistent stability allowed clinicians to periodically monitor and perform a complete neurological, neurocognitive, and neuroimaging assessment, which finally confirmed the underlying organic condition to ES, otherwise fully/partially masked by psychotic symptomatology. These specialistic evaluations led to a diagnosis of ES secondary to DLB, which became more clinically evident through a dramatic decline in cognitive functions and loss of autonomy in everyday life.

However, this treatment option with LAI APs is not advisable when symptoms of ES appear in elderly patients with no history of previous psychotic symptoms and/or disorder, where an underlying neurological cause should always be suspected and carefully investigated before treatment choice. In this case, the late diagnosis of DLB resulted in the patient being treated with AP drugs despite her neuroleptic sensitivity, ultimately leading to extrapyramidal symptoms. This risk is especially prominent with LAI formulations, which should have been avoided entirely if the final diagnosis had been known, because they expose the patients not only to the occurrence of mild neuroleptic sensitivity, as experienced by this patient, but also to severe idiosyncratic forms that can result in a neuroleptic malignant syndrome [16]. DLB should have been promptly investigated as the patient presented with a pattern compatible with psychiatric onset DLB [15], characterised by late-onset psychosis with hallucinations in visual and other modalities and systematised delusions (ES), in the presence of anxiety and depression, severe enough to require hospitalisation. Furthermore, the patient presented cognitive impairment, despite arduous to explore given the psychotic symptoms, and she presented parkinsonism after a period of treatment with neuroleptics in LAI formulation, all features that contributed to corroborate the hypothesis of an underlying DLB. Unlike previously reported cases of ES in a DLB [18,19], the diagnosis was delayed, as it mostly relied upon proper identification of neurocognitive symptoms in a patient with poor explorability of her mental state due to psychiatric-onset DLB. It could have been obtained sooner and with more precision in the observation period through the use of indicative biomarkers (earlier DaT imaging, myocardial scintigraphy, and polysomnography) [13,15,18,19].

Finally, a patient-tailored, multidisciplinary approach associated with continuity of care in specialistic settings and close periodic follow-up visits may ensure a definitive diagnosis and allow the patient to prompt treatment for DLB.
